# Association of VEGF Genetic Polymorphisms with Recurrent Spontaneous Abortion Risk: A Systematic Review and Meta-Analysis

**DOI:** 10.1371/journal.pone.0123696

**Published:** 2015-04-20

**Authors:** Xinghua Xu, Chigang Du, Huihui Li, Jing Du, Xue Yan, Lina Peng, Guangyao Li, Zi-Jiang Chen

**Affiliations:** 1 Department of Gynecology and Obstetrics, Liaocheng People's Hospital, Liaocheng, 252000, China; 2 Center for Reproductive Medicine, Shandong Provincial Hospital Affiliated to Shandong University, Jinan, 250021, China; 3 Shandong Provincial Key Laboratory of Reproductive Medicine, Jinan, 250021, China; 4 National Research Center for Assisted Reproductive Technology and Reproductive Genetics, Jinan, 250021, China; 5 The Key laboratory for Reproductive Endocrinology of Ministry of Education, Jinan, 250021, China; 6 Department of Neurosurgery, Liaocheng People's Hospital, Liaocheng, 252000, China; 7 Center for Reproductive Medicine, Ren Ji Hospital, School of Medicine, Shanghai Jiao Tong University, Shanghai, 200127, China; 8 Shanghai Key Laboratory for Assisted Reproduction and Reproductive Genetics, Shanghai, 200127, China; 9 Department of Gynecology and Obstetrics, Tianjin third central hospital, Tianjin, 300170, China; 10 Department of Hematology, Liaocheng People's Hospital, Liaocheng, 252000, China; State Key Laboratory of Reproductive Biology, Institute of Zoology, Chinese Academy of Sciences, CHINA

## Abstract

**Background:**

Studies of the associations between the genetic polymorphisms of the vascular endothelial growth factor (VEGF) gene and recurrent spontaneous abortion (RSA) have revealed conflicting results. The present meta-analysis was performed to provide a more precise estimation of these relationships and to explore potential sources of heterogeneity that may have influenced the reported disparities.

**Methods:**

An extensive literature search for relevant studies was conducted on PubMed, Embase, and The Cochrane Library through June 6, 2014. Crude odds ratio (OR) with 95% confidence intervals were calculated.

**Results:**

10 case-control studies including 1,832 RSA patients and 2,271 healthy controls were identified. Meta-analysis indicated that rs1570360, rs3025039, rs2010963, and rs3025020 polymorphisms in the VEGF gene correlated with elevated RSA risk. The rs1570360 variant was statistically significantly relevant to RSA risk among non-Asian populations. Interestingly, the rs3025039 variant was statistically significantly relevant to RSA risk among Asian populations.

**Conclusions:**

The current meta-analysis indicates that rs1570360, rs3025039, rs2010963, and rs3025020 polymorphisms increase RSA susceptibility. Moreover, rs1570360 and rs3025039 polymorphisms may play various roles in RSA susceptibility in various geographic groups.

## Introduction

Recurrent spontaneous abortion (RSA), defined as three or more consecutive pregnancy losses before the 20th weeks of gestation, is a significant reproductive problem. Around 1–3% of couples trying to conceive experience RSA[1,2]. Until now, various factors have been identified that influence miscarriage, including uterine pathologies, endocrine dysfunctions, autoimmune diseases, acquired and inherited thrombophilia, as well as nutritional and environmental factors[3,4]. In up to 50% of RSA patients, however, the exact underlying etiology remains undetermined.

Vascular endothelial growth factor (VEGF) is a specific mitogen and survival factor for endothelial cells and also a prime mediator of angiogenesis and vasculogenesis under physiological and pathological conditions[5–7]. A central role of VEGF in fetal and placental angiogenesis has been provided from gene knockout studies[8–10]. During early gestation, VEGF is essential for the maturation of oocytes, the proliferation of trophoblasts, the implantation and development of the embryo, the angiogenesis of the placenta, and the growth of maternal and fetal blood vessels in the uterus[11,12]. Based on the above findings, it is plausible that vascular endothelial growth factor may be involved in the pathogenesis of RSA in women.

The human VEGF gene is located on chromosome 6p21.3 and consists of eight exons and seven introns, spanning approximately 14 kb[13,14].As we all know in RSA patients, several commonly studied SNPs in the VEGF gene have been identified, such as −2578C/A (rs699947), −1154G/A (rs1570360), −634G/C (rs2010963), +936C/T (rs3025039), and −583T/C (rs3025020). Many previous studies have revealed that functional polymorphisms of the VEGF gene may play a role in the pathogenesis of RSA[15–21]. Building on these foundational observations, several studies have focused on the association between the SNPs of the VEGF gene and the presence of RSA, but these studies have yielded inconsistent results[12,22,23]. A previous meta-analysis investigating this relationship was conducted in 2012[24]. In view of 8 eligible studies, the results suggested that VEGF gene −2578C/A (rs699947) and −1154G/A(rs1570360) polymorphisms were not significantly associated with the risk of RSA, whereas −634G/C (rs2010963) and+936C/T (rs3025039) polymorphisms were associated with the risk of RSA under specific genetic models. Nevertheless, careful examination of the data used in that study revealed a noteworthy inconsistency of diagnostic criteria. The specific choice of phenotype for the cases may define the exact hypothesis to be tested, and applying strict clinical criteria for ascertainment is necessary to ensure a homogeneous set of cases[25]. The inclusion criteria of RSA from the studies reported by Lee et al. and Coulam et al. were defined as patients being diagnosed with at least two consecutive spontaneous abortions[26,27]. And the RSA patients from the other 6 studies were diagnosed with three or more consecutive pregnancy losses before the 20th weeks of gestation. The inconsistency of diagnostic criteria would lead to unconvincing and unreliable results in associating VEGF genetic polymorphisms to RSA risk.

Several more replication studies were performed in the past two years to reevaluate the effect of VEGF gene polymorphisms on RSA offered some new data and diverse conclusions [16,17,19,20]. Comparing with the previous meta-analysis, more samples and more SNPs (−583T/C increased) were involved in our study.

To more clearly address the question of an association between these genetic variations of the VEGF gene and RSA, we conducted the current systematic review and meta-analysis with much stricter entry criteria to clarify such inconsistencies and to identify potential sources of heterogeneity that might confound the conclusions.

## Materials and Methods

### Search Strategy

Literature searches were performed by 2 investigators and the final search strategies were performed with agreement. An extensive literature search for relevant studies was conducted on PubMed, Embase, and The Cochrane Library from inception through June 6, 2014. We used the following keywords and MeSH terms: (“vascular endothelial growth factor” OR “VEGF”) AND (“recurrent spontaneous miscarriage” OR “recurrent spontaneous abortion” OR “recurrent pregnancy loss”). There were no language restrictions. Any clearly irrelevant studies, editorials, and review articles were excluded. Duplicate publications were considered only once. The remaining articles were carefully read in their entirety to determine whether they contained information on the topic of interest. Furthermore, the reference sections of review articles and other relevant studies were searched manually for additional eligible studies.

### Selection Criteria

To be included in the analysis, studies had to meet the following criteria: (1) independent case-control or cohort studies; (2) inclusion of both RSA cases and non- RSA controls; (3) examination of the association between VEGF genetic polymorphisms and RSA risk; (4) inclusion of adequate data to calculate the effect size of allele or genotype frequencies; and (5) genotype distribution in healthy controls conforming to Hardy-Weinberg equilibrium(HWE). Accordingly, the following exclusion criteria were also used: (1) no healthy control population; (2) genotype frequency unavailable; (3) non-conformity with the criteria for RSA; and (4) duplication of previous publications. Any disagreements were resolved by discussion until a consensus was reached.

### Data Extraction

2 authors independently extracted data from eligible studies by using a standardized form. The following information was collected prospectively: gene polymorphism, the first author’s name, year of publication, country of origin, diagnostic criteria for RSA, genotype number in cases and controls, genotype method, and the p-value of Hardy-Weinberg equilibrium (HWE) in controls. In cases of conflicting evaluations, disagreements on inconsistent data from the eligible studies were resolved through discussion and careful reexamination of the full text by the authors.

### Quality assessment

The quality of the included studies was independently assessed by 2 authors using the criteria modified from the previous report[28]. The criteria were as follows:

1. Description of the cases and controls (adequate, inadequate);
2. Assessment and validation of miscarriage in the cases (adequate, inadequate, not stated). Adequate validation would include confirmation by pathological examination or scan; inadequate validation would include recollection of the patient as the only evidence or a biochemical pregnancy;
3. Description of the laboratory procedures (adequate, inadequate);
4. Level of exclusion of confounding factors inpatients (not described, inadequate, adequate). “Adequate” refers to the elimination of the proven causes of recurrent miscarriage (chromosomal abnormalities in the couples, antiphospholipid antibodies, mullerian abnormalities of the uterus, protein C/S/antithrombin-III deficiency);
5. Equal assessment for confounding factors in the case and control groups (equal, unequal, not stated).

### Statistical Analysis

Allele frequencies at VEGF gene polymorphisms from each study were determined by the allele counting method. HWE was assessed in each study using the goodness-of-fit test (chi-square test) in control groups. Crude odds ratios (ORs) with 95% confidence intervals (CIs) were used to assess the strength of the association between the VEGF polymorphisms and the risk of RSA in codominant, homozygous, heterozygous, dominant, and recessive genetic models based on the genotype frequencies in cases and controls. Heterogeneity was measured using Cochran's Q test and I^2^ statistic[29]. The corresponding p-value of the Q statistic below 0.05 or I^2^>50% was considered significant heterogeneity. If there was a statistical difference in terms of heterogeneity, pooled ORs were calculated using a fixed-effects model (I^2^≤50%) or a random-effects model (I^2^>50%). To statistically assess the publication bias of studies, funnel plots and Egger’s tests were carried out[30]. To consider potential geographic variation, we performed subgroup analysis stratified by geographic position. All statistical analyses were performed by Stata version 12 (StataCorp LP, College Station, TX, USA) and Review Manager 5.0. All p-values were two-sided and p <0.05 was defined as significant.

## Results

### Study Characteristics

**Fig 1:**
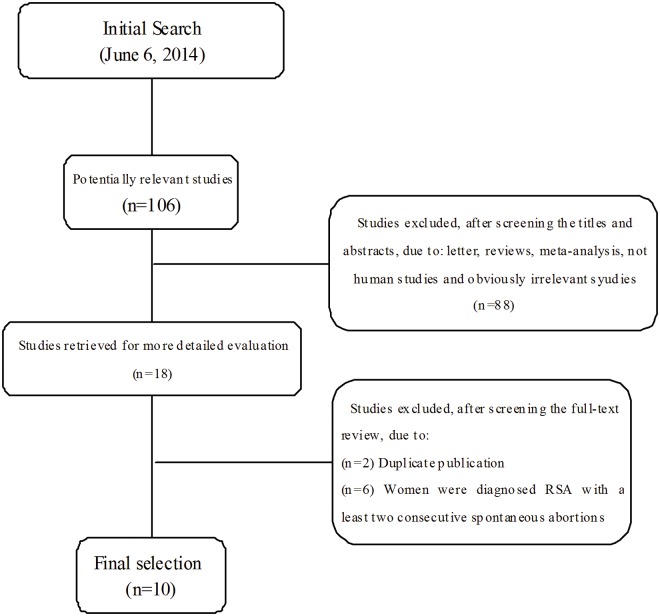
Flow chart of literature search and study selection.

A total of 106 articles relevant to the searched keywords were initially identified. Of these articles, 18 were selected as potentially relevant studies after reading the titles and abstracts. Full texts were then reviewed for a more detailed evaluation. As a result, 10 studies met the entry criteria and were involved in this meta-analysis[12,15–23]. The main reasons for exclusion were as follows: 2 papers were duplicate publications[31,32], and the other 6 excluded studies diagnosed RSA with at least two consecutive spontaneous abortions[26,27,33–36]. Searching reference lists produced no other eligible publications. The study selection process is shown in Fig 1.

A total of 4,103 subjects were involved in this meta-analysis, including 1,832 RSA patients and 2,271 healthy controls. Overall, 4 of these studies were conducted in Asia and the other 6 studies were not conducted in Asia (Greece, USA, Brazil, Tunisian, Bahrain, and Arabian, respectively). Among all the SNPs of the VEGF gene addressed, −2578C/A (rs699947), −1154G/A (rs1570360), −634G/C (rs2010963), +936C/T (rs3025039), and −583T/C (rs3025020) were the most common. DNA samples used for determination of VEGF genetic polymorphisms were extracted from blood in all included studies. Methods used for genotyping include polymerase chain reaction-restriction fragment length polymorphism (PCR-RELP), Taqman real-time PCR, and MassArray. Genotype distributions among the controls of all studies were consistent with Hardy-Weinberg equilibrium except for only one study with −1154G/A (rs1570360) genotype[15]. Detailed characteristics of the included studies are summarized in Table 1.

**Table 1:** Main characteristics of the studies included in the meta-analysis.

Gene polymorphism	Author	Year	Country	DiagnosticCriteria(numbers of consecutive pregnancy losses)	Genotype ^a^		Genotype method	P for HWE^b^	Quality Assessment
					Case	Control			
rs2010963	Papazoglou	2005	Greece	three or more	14/22/16	29/35/18	PCR–RFLP	0.237	1: adequate; 2: not stated;3: adequate;4: adequate; 5: not stated
	Eller	2011	USA	three or more	36/45/15	88/73/18	TaqMan	0.62	1: adequate; 2: not stated;3: adequate;4: adequate; 5: unequal
	Traina	2011	Brazil	three or more	23/37/17	27/47/11	PCR	0.177	1: adequate; 2: not stated;3: adequate;4: adequate; 5: not stated
	Magdoud	2012	Tunisian	three or more	131/130/43	154/166/51	TaqMan	0.558	1: adequate; 2: not stated;3: adequate;4: adequate; 5: unequal
	Almawi	2013	Bahrain	three or more	96/137/63	127/127/51	TaqMan	0.0502	1: adequate; 2: not stated;3: adequate;4: adequate; 5: unequal
rs3025020	Al-Khateeb	2011	Arabian	three or more	49/57/67	23/106/119	TaqMan	0.931	1: adequate; 2: not stated;3: adequate;4: adequate; 5: unequal
	Almawi	2013	Bahrain	three or more	82/95/119	27/130/148	TaqMan	0.838	1: adequate; 2: not stated;3: adequate;4: adequate; 5: unequal
	li	2013	China	three or more	62/96/69	33/105/94	MassARRAY	0.647	1: adequate; 2: not stated;3: adequate;4: adequate; 5: not stated
rs3025039	Papazoglou	2005	Greece	three or more	1/16/35	1/17/64	PCR–RFLP	0.914	1: adequate; 2: not stated;3: adequate;4: adequate; 5: not stated
	Aggarwal	2011	Indian	three or more	6/52/142	1/35/164	RFLP	0.549	1: adequate; 2: not stated;3: adequate;4: adequate; 5: not stated
	Eller	2011	USA	three or more	1/29/67	6/45/127	TaqMan	0.423	1: adequate; 2: not stated;3: adequate;4: adequate; 5: unequal
	Traina	2011	Brazil	three or more	1/20/59	1/27/101	PCR	0.578	1: adequate; 2: not stated;3: adequate;4: adequate; 5: not stated
	Magdoud	2012	Tunisian	three or more	10/87/207	6/66/299	TaqMan	0.294	1: adequate; 2: not stated;3: adequate;4: adequate; 5: unequal
	Almawi	2013	Bahrain	three or more	5/59/232	8/65/232	TaqMan	0.192	1: adequate; 2: not stated;3: adequate;4: adequate; 5: unequal
	li	2013	China	three or more	21/78/128	10/63/159	MassARRAY	0.249	1: adequate; 2: not stated;3: adequate;4: adequate; 5: not stated
rs699947	Papazoglou	2005	Greece	three or more	16/21/15	21/34/27	PCR–RFLP	0.132	1: adequate; 2: not stated;3: adequate;4: adequate; 5: not stated
	Aggarwal	2011	Indian	three or more	23/74/103	17/67/116	PCR	0.111	1: adequate; 2: not stated;3: adequate;4: adequate; 5: not stated
	Eller	2011	USA	three or more	15/43/38	37/96/44	TaqMan	0.25	1: adequate; 2: not stated;3: adequate;4: adequate; 5: unequal
	Magdoud	2012	Tunisian	three or more	48/152/104	67/182/122	TaqMan	0.951	1: adequate; 2: not stated;3: adequate;4: adequate; 5: unequal
	Almawi	2013	Bahrain	three or more	53/117/126	54/135/116	TaqMan	0.181	1: adequate; 2: not stated;3: adequate;4: adequate; 5: unequal
	li	2013	China	three or more	14/86/127	8/78/146	MassARRAY	0.536	1: adequate; 2: not stated;3: adequate;4: adequate; 5: not stated
rs1570360	Papazoglou	2005	Greece	three or more	15/19/18	12/28/42	PCR–RFLP	0.055	1: adequate; 2: not stated;3: adequate;4: adequate; 5: not stated
	Aggarwal	2011	Indian	three or more	32/48/120	18/47/135	PCR	0	1: adequate; 2: not stated;3: adequate;4: adequate; 5: not stated
	Eller	2011	USA	three or more	11/27/55	18/75/85	TaqMan	0.808	1: adequate; 2: not stated;3: adequate;4: adequate; 5: unequal
	Su	2011	China	three or more	1/16/36	7/39/124	TaqMan	0.094	1: adequate;2: not stated;3: adequate;4: adequate; 5: not stated
	Xing	2011	China	three or more	10/81/200	31/118/155	TaqMan	0.613	1: adequate;2: not stated;3: adequate; 4: adequate; 5: equal
	Magdoud	2012	Tunisian	three or more	15/126/230	16/9/13	TaqMan	0.662	1: adequate; 2: not stated;3: adequate;4: adequate; 5: unequal
	Almawi	2013	Bahrain	three or more	33/119/153	16/85/126	TaqMan	0.18	1: adequate; 2: not stated;3: adequate;4: adequate; 5: unequal
	li	2013	China	three or more	16/85/126	10/77/145	MassARRAY	0.956	1: adequate; 2: not stated;3: adequate;4: adequate; 5: not stated

^a^Genotype, for rs2010963, GG/GC/CC; for rs3025020, TT/CT/CC; for rs3025039, TT/CT/CC, for rs699947, AA/AC/CC, for rs833061, CC/CT/TT; and for rs1570360, AA/GA/GG.

^b^ HWE, Hardy-Weinberg equilibrium.

PCR, polymerase chain reaction; RFLP, restriction fragment length polymorphism.

### Meta-Analysis Results

Overall, the ORs and 95% CIs of RSA were considered under codominant, homozygous, heterozygous, dominant, and recessive genetic models. A summary of all the meta-analyses findings of the associations between VEGF genetic polymorphisms and RSA risk is provided in Table 2.

**Table 2:** Meta-analysis results for the five studied polymorphisms and RSA risk.

Gene polymorphism	Inherited model	Heterogeneity-test		Analysis model ^a^	Pooled OR (95% CI)	P
		P for Q test	I^2^(%)			
rs2010963	Codominant(C vs. G)	0.22	29	FEM	1.16 [1.03, 1.31]	0.01
	Homozygous(CC vs. GG)	0.42	0	FEM	1.36 [1.06, 1.74]	0.02
	Heterozygous(CC vs. GC)	0.87	0	FEM	1.20 [0.94, 1.52]	0.14
	Dominant(CC+GC vs. GG)	0.25	25	FEM	0.85 [0.72, 1.01]	0.06
	Recessive(CC vs. GC+GG)	0.68	0	FEM	0.79 [0.63, 0.98]	0.04
rs3025020	Codominant(C vs. T)	0.76	0	FEM	0.57 [0.49, 0.67]	<0.001
	Homozygous(CC vs. TT)	0.05	0	FEM	0.30 [0.22, 0.41]	<0.001
	Heterozygous(TT vs. CT)	0.11	55	REM	3.20 [2.02, 5.07]	<0.001
	Dominant(TT+CT vs. CC)	0.92	0	FEM	0.68 [0.55, 0.84]	0.0004
	Recessive(TT vs. CT+CC)	0.19	39	FEM	3.22 [2.43, 4.27]	<0.001
rs3025039	Codominant(C vs. T)	0.02	61	REM	0.72 [0.56, 0.93]	0.01
	Homozygous(CC vs. TT)	0.20	30	FEM	0.57 [0.36, 0.90]	0.02
	Heterozygous(TT vs. CT)	0.58	0	FEM	1.22 [0.75, 1.98]	0.43
	Dominant(TT+CT vs. CC)	0.06	51	REM	0.69 [0.53, 0.89]	<0.005
	Recessive(TT vs. CT+CC)	0.28	20	FEM	1.60 [1.01, 2.54]	0.05
rs699947	Codominant(C vs. A)	0.04	58	REM	0.99 [0.82, 1.20]	0.92
	Homozygous(CC vs. AA)	0.11	44	FEM	1.04 [0.81, 1.32]	0.78
	Heterozygous(AA vs. CA)	0.82	0	FEM	1.05 [0.82, 1.34]	0.70
	Dominant(AA+CA vs.CC)	0.04	58	REM	1.03 [0.79, 1.33]	0.85
	Recessive(AA vs. CA+CC)	0.42	0	FEM	1.01 [0.81, 1.27]	0.91
rs1570360	Codominant(G vs. A)	0.002	72	REM	0.88 [0.69, 1.12]	0.029
	Homozygous(GG vs. AA)	0.02	61	REM	0.72 [0.43, 1.21]	0.21
	Heterozygous(GG vs. GA)	0.02	60	REM	0.98 [0.75, 1.28]	0.86
	Dominant(GG+GA vs. AA)	0.07	48	FEM	1.49 [1.12, 1.96]	0.005
	Recessive(GG vs. GA+AA)	0.005	68	REM	0.91 [0.69, 1.21]	0.52

^a^ REM: random-effect model. FEM: fixed-effect model.

P-value of overall effect.

### −1154G/A (rs1570360) and RSA risk

**Fig 2:**
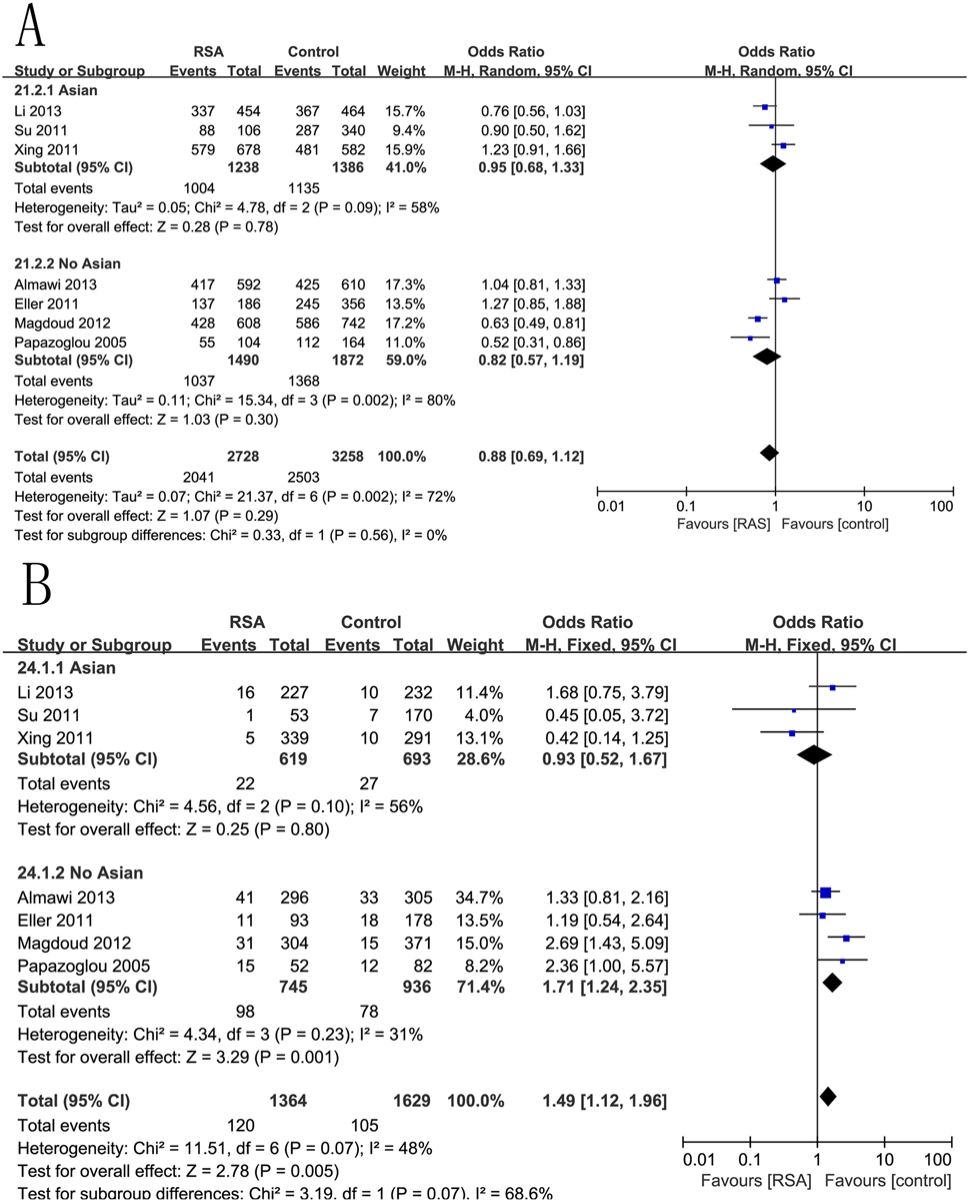
Forest plots for the associations between rs1570360 polymorphism in the VEGF gene and RSA risk using. (A) codominant genetic models (G vs. A); (B) (D) dominant genetic models (GG+GA vs. AA).

A variant −1154G/A (rs1570360) was the most studied polymorphism in the VEGF gene, with 8 data sets supporting an increased risk of RSA. We excluded one study that was not in good fitness with HWE[15]. There was significant association between −1154G/A (rs1570360) polymorphism and RSA risk in the contrast of G allele versus A allele, with a pooled OR = 0.88 (95% CI 0.69–1.12, p = 0.029) (Fig 2A). We found similar effect size in the dominant (GG+GA vs. AA) model, with OR = 1.49(95% CI 1.12–1.96, p = 0.005) (Fig 2B). However, no significant associations were found in the homozygous, heterozygous, or recessive models. The results were as follows: GG vs. AA (OR = 0.72, 95%CI 0.43–1.21, p = 0.21), GG vs. GA (OR = 0.98, 95%CI 0.75–1.28, p = 0.86), GG vs. GA+AA (OR = 0.91, 95%CI 0.69–1.21, p = 0.52) (Table 2).

To establish the effects of heterogeneity on the results, a subgroups analysis of geographic position was performed. Stratification by geographic position indicated that the polymorphism of rs1570360 was significantly associated with RSA for non-Asians rather than Asians under codominant and dominant genetic models. The pooled ORs were 0.82 (95%CI 0.57–1.19, p = 0.03), and 0.95 (95%CI 0.68–1.33, p = 0.78) for non-Asians and Asians, respectively, under the codominant model (G vs. A). Similar results were also detected under the dominant genetic model. The results of this subgroup analysis are displayed in Table 3 and Fig 2.

**Table 3:** Results of subgroups analysis.

Gene polymorphism	Inherited model	Subgroup	Heterogeneity-test		Analysis model ^a^	Pooled OR (95% CI)	P
			P for Q test	I^2^ (%)			
rs1570360	Codominant (G vs. A)	Asian	0.009	58	REM	0.95 [0.68, 1.33]	0.78
	Codominant (G vs. A)	No-Asian	0.002	80	REM	0.82 [0.57, 1.19]	0.03
	Homozygous(GG vs. AA)	Asian	0.08	61	REM	1.22 [0.39, 3.81]	0.73
	Homozygous(GG vs. AA)	No-Asian	0.04	64	REM	0.58 [0.32, 1.04]	0.07
	Heterozygous (GG vs. GA)	Asian	0.32	13	REM	0.91 [0.70, 1.19]	0.5
	Heterozygous (GG vs. GA)	No-Asian	0.0006	76	REM	1.05 [0.66, 1.68]	0.84
	Dominant (GG+GA vs. AA)	Asian	0.1	56	FEM	0.93 [0.52, 1.67]	0.8
	Dominant (GG+GA vs. AA)	No-Asian	0.23	31	FEM	1.71 [1.24, 2.35]	0.001
	Recessive (GG vs. GA+AA)	Asian	0.18	42	REM	0.92 [0.66, 1.27]	0.61
	Recessive (GG vs. GA+AA)	No-Asian	0.002	80	REM	0.91 [0.56, 1.46]	0.69
	Codominant (C vs. T)	Asian	0.65	0	REM	0.58 [0.45, 0.75]	<0.0001
	Codominant (C vs. T)	No-Asian	0.02	65	REM	0.80 [0.57, 1.13]	0.32
	Dominant (TT+CT vs. CC)	Asian	0.75	0	REM	0.57 [0.42, 0.77]	0.0002
	Dominant (TT+CT vs. CC)	No-Asian	0.04	60	REM	0.76 [0.53, 1.08]	0.13
	Recessive (GG vs. GA+AA)	Asian	0.18	42	REM	0.92 [0.66, 1.27]	0.61
	Recessive (GG vs. GA+AA)	No-Asian	0.002	80	REM	0.91 [0.56, 1.46]	0.69
rs3025039	Codominant (C vs. T)	Asian	0.65	0	REM	0.58 [0.45, 0.75]	<0.0001
	Codominant (C vs. T)	No-Asian	0.02	65	REM	0.80 [0.57, 1.13]	0.32
	Dominant (TT+CT vs. CC)	Asian	0.75	0	REM	0.57 [0.42, 0.77]	0.0002
	Dominant (TT+CT vs. CC)	No-Asian	0.04	60	REM	0.76 [0.53, 1.08]	0.13

^a^ REM: random-effect model. FEM: fixed-effect model.

P-value of overall effect.

### +936C/T (rs3025039) and RSA Risk

**Fig 3:**
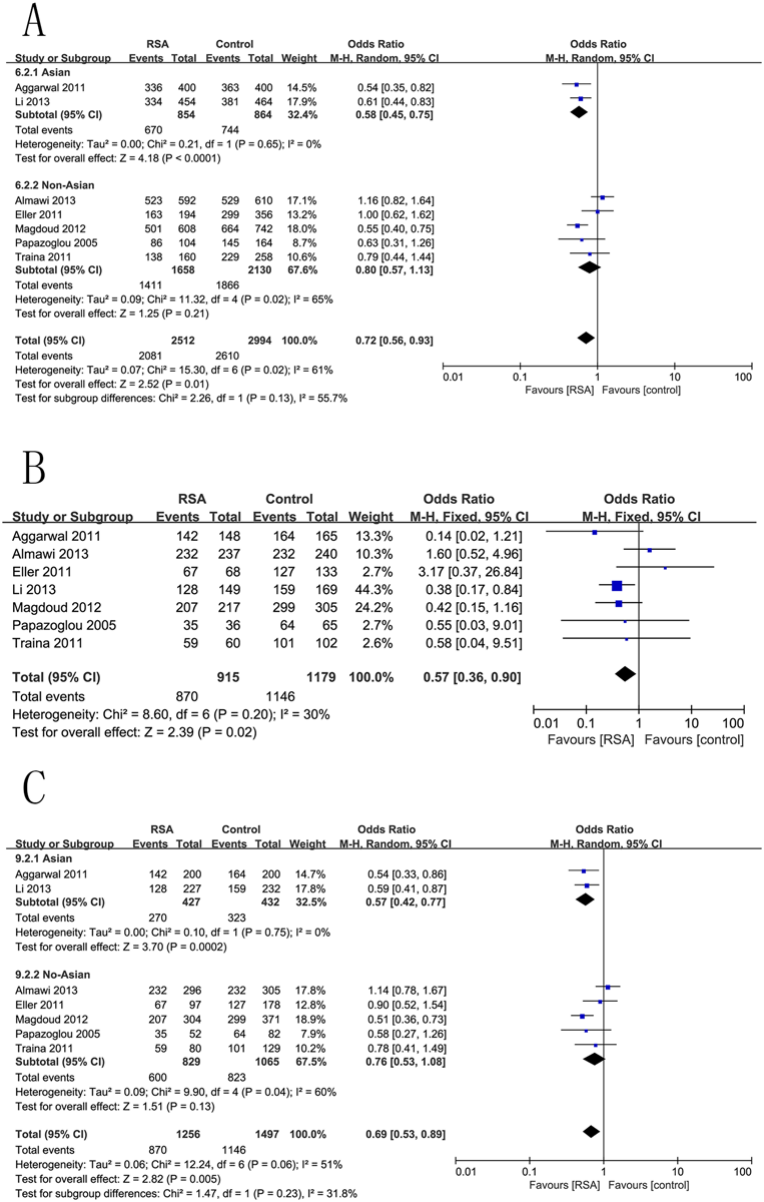
Forest plots for the associations between rs3025039 polymorphisms in the VEGF gene and RSA risk using. (A) codominant genetic models (C vs. T); (B) homozygous genetic models (CC vs. TT); and (C) dominant genetic models (TT+CT vs. CC).

The combined results of all analyses showed that the +936C/T (rs3025039) allele increased the risk of RSA in the codominant model (C vs. T: OR = 0.72, 95%CI 0.56–0.93, p = 0.01) (Fig 3A), homozygous comparison (CC vs. TT: OR = 0.57, 95%CI 0.36–0.90, p = 0.02) (Fig 3B) and the dominant model (TT+CT vs. CC: OR = 0.69, 95%CI 0.53–0.89, p< 0.005) (Fig 3C), but no significant associations were found in the heterozygous and recessive models. The results were as follows: TT vs. CT (OR = 1.22, 95%CI 0.75–1.98, p = 0.43), TT vs. CT+CC (OR = 1.60, 95%CI1.01–2.54, p = 0.05) (Table 2).

Stratification by geographic position indicated that the polymorphism of rs3025039 was significantly associated with RSA for Asians rather than non-Asians under codominant and dominant genetic models. The pooled ORs were 0.52 (95%CI 0.45–0.75, p<0.0001), and 0.80 (95%CI 0.57–1.13, p = 0.32) for Asians and non-Asians, respectively, under the codominant model (C vs. T). Similar results were also detected under the dominant genetic model. The results of this subgroup analysis are displayed in Table 3 and Fig 3.

### −2578C/A (rs699947) and RSA Risk

No associations were observed between −2578C/A (rs699947) polymorphism and RSA risk using all the genetic models. The results were as follows: C vs. A (OR = 0.99, 95%CI 0.82–1.20, p = 0.92), CC vs. AA (OR = 1.04, 95%CI 0.81–1.32, p = 0.78), AA vs. CA (OR = 1.05, 95%CI 0.82–1.34, p = 0.70), AA+CA vs. CC (OR = 1.03, 95%CI 0.79–1.33, p = 0.85), and AA vs. CA+CC (OR = 1.01, 95%CI0.81–1.27, p = 0.91) (Table 2).

### −634G/C (rs2010963) and RSA Risk

**Fig 4:**
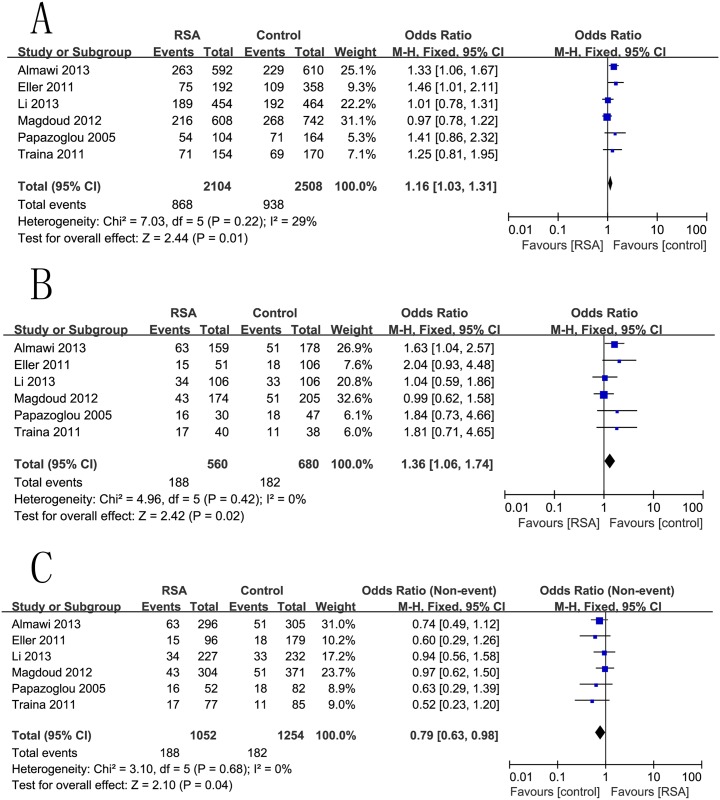
Forest plots for the associations between rs2010963 polymorphisms in the VEGF gene and RSA risk using. (A) codominant genetic models (C vs. G); (B) homozygous genetic models (CC vs. GG); and (C) recessive genetic models (CC vs. GC+GG).

The combined results of all analyses showed that the −634G/C (rs2010963) allele increased the risk of RSA in the codominant model (C vs. G: OR = 1.16, 95%CI 1.03–1.31, p = 0.01) (Fig 4A), homozygous comparison (CC vs. GG: OR = 1.36, 95%CI 1.06–1.74, p = 0.02) (Fig 4B), and the recessive model (CC vs. GC+GG: OR = 0.79, 95%CI 0.63–0.98, p = 0.04) (Fig 4C), but no significant associations were found in the heterozygous and dominant models where the results were as follows: CC vs. GC (OR = 1.20, 95%CI 0.94–1.52, p = 0.14), and CC+GC vs. GG (OR = 0.85, 95%CI 0.72–1.01, p = 0.06) (Table 2).

### −583T/C (rs3025020) and RSA Risk

**Fig 5:**
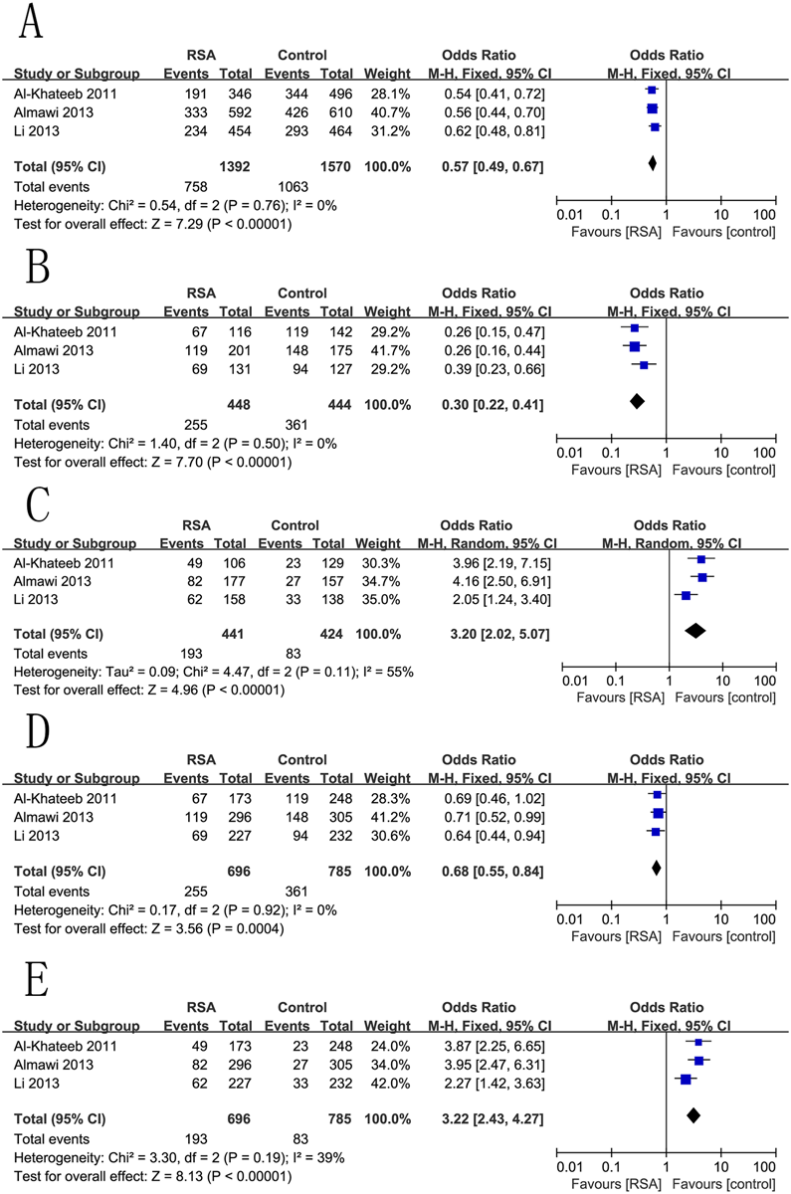
Forest plots for the associations between rs3025020 polymorphisms in the VEGF gene and RSA risk using. (A) codominant genetic models (C vs. T); (B) homozygous genetic models (CC vs. TT); (C) heterozygous genetic models (TT vs. CT); (D) dominant genetic models (TT+CT vs. CC); and (E) recessive genetic models (TT vs. CT+CC).

An initial meta-analysis using the fixed-effects model was performed, which comprised all the participants mentioned above from diverse ethnic groups. There was significant association between −583T/C (rs3025020) polymorphism and RSA risk in the contrast of C allele versus T allele, with a pooled OR = 0.57 (95% CI 0.49–0.67, p <0.001) (Fig 5A). We found similar effect sizes in homozygous (CC vs. TT), heterozygous (TT vs. CT), dominant (TT+CT vs. CC), and recessive (TT vs. CT+CC) models, with OR = 0.30 (95% CI 0.22–0.41, p <0.001) (Fig 5B), OR = 3.20 (95% CI 2.02–5.07, p <0.001) (Fig 5C), OR = 0.68 (95% CI 0.55–0.84, p = 0.0004) (Fig 5D), and OR = 3.22 (95% CI 2.43–4.27, p <0.001) (Fig 5E), respectively.

### Sensitivity Analysis

**Fig 6:**
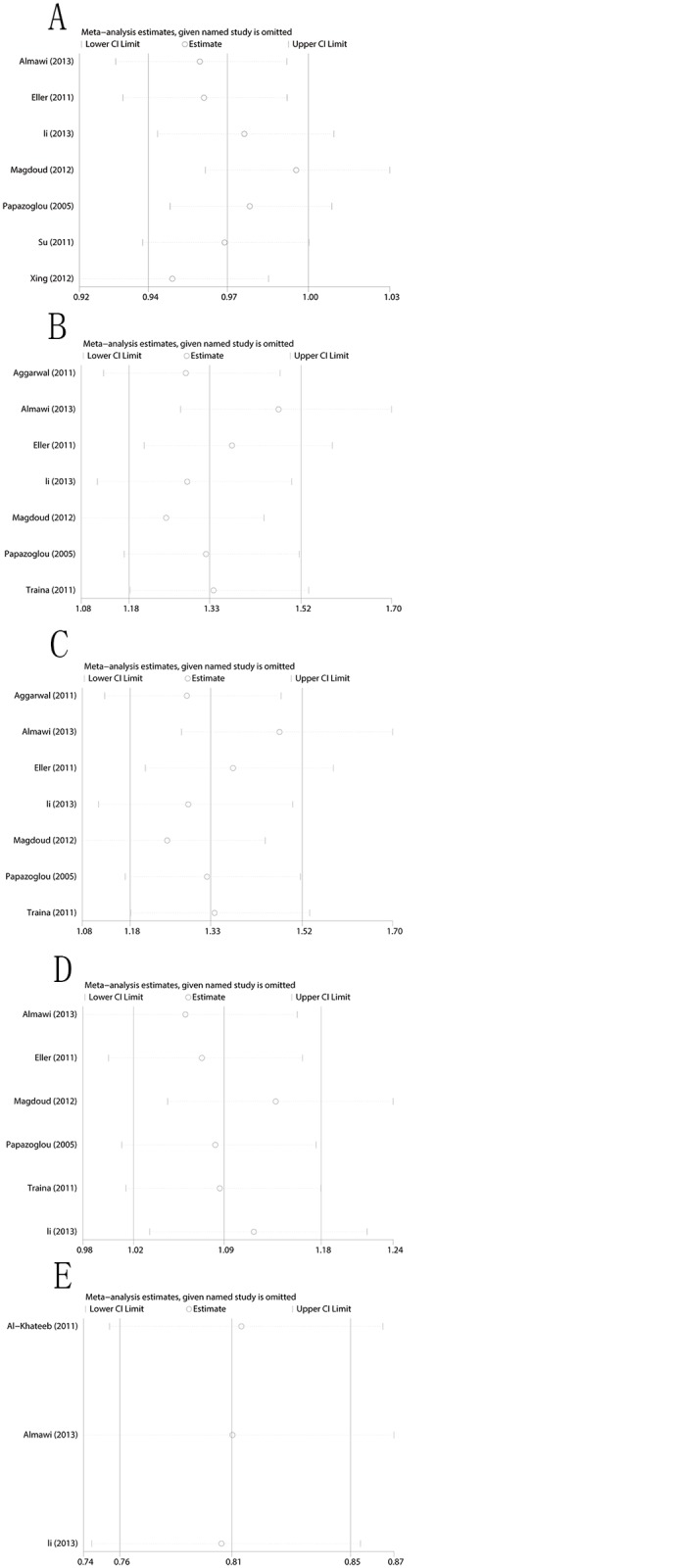
Sensitivity analysis for the associations between polymorphisms in the VEGF gene and RSA risk. (A) Sensitivity analysis for rs1570360 and RSA risk; (B) Sensitivity analysis for rs3025039 and RSA risk; (C) Sensitivity analysis for rs699947 and RSA risk; (D) Sensitivity analysis for rs2010963 and RSA risk; and (E) Sensitivity analysis for rs3025020 and RSA risk.

A sensitivity analysis was conducted to assess the influence of each individual study on the pooled OR by removing each study in turn. The results demonstrated no evidence of any individual study having excessive influence on the pooled OR under the dominant model (Fig 6).

### Publication Bias

**Fig 7:**
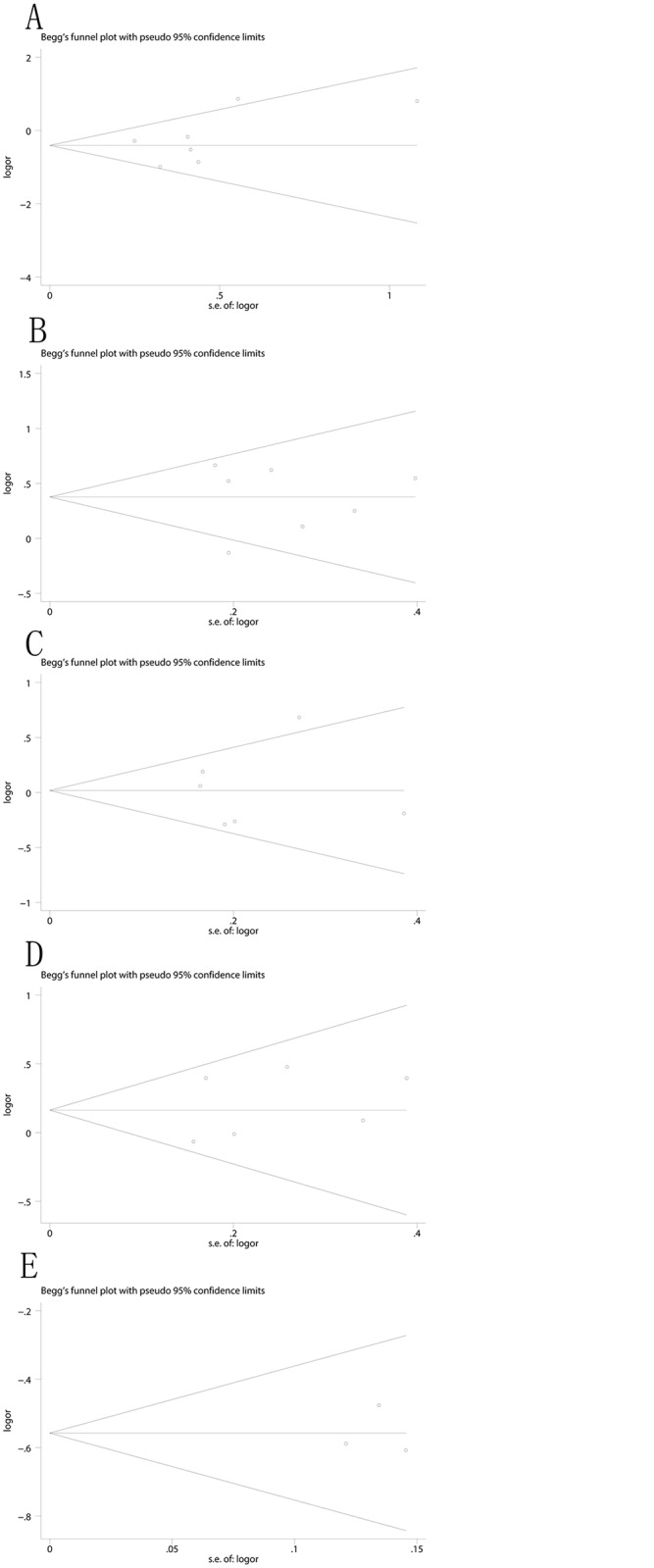
Funnel plot for the associations between polymorphisms in the VEGF gene and RSA risk. (A) rs1570360(Egger test: t = 1.10, p = 0.321); (B) rs3025039 (Egger test: t = -0.16, p = 0.883); (C) rs699947 (Egger test: t = 0.18, p = .0867); (D) rs2010963 (Egger test: t = 0.69, p = 0.526); and (E) rs3025020(Egger test: t = 0.02, p = 0.988).

Funnel plots and Egger's linear regression tests were performed to assess the publication bias of included studies. The shapes of the funnel plots seemed symmetrical (Fig 7) suggesting that there were no significant publication bias under the dominant model. The Egger's test also did not display strong statistical evidence of publication bias (rs1570360: t = 1.10, p = 0.321; rs3025039: t = -0.16, p = 0.883; rs699947: t = 0.18, p = 0.867; rs2010963: t = 0.69, p = 0.526; rs3025020: t = 0.02, p = 0.988) (Fig 7).

## Discussion

As a major factor in angiogenesis, VEGF has attracted attention because of its involvement in abnormalities of embryo development, the development of cancers, and cerebrovascular and cardiovascular diseases. Furthermore, there is convincing evidence of a crucial role of VEGF in fetal and placental angiogenesis [8]. Increasing evidence shows that aberrations in vascular formation and/or function contribute to RSA [37].Moreover, first trimester trophoblast VEGF expression was weaker in placental samples from RSA cases than in gestational age-matched normal placenta [38,39]. Increased blood vessel density in deciduas parietalis was related with spontaneous human first trimester abortion[40]. Based on the above, VEGF may be involved in the pathogenesis of RSA.

VEGF gene polymorphisms have been demonstrated to be relevant to the change of VEGF protein expression[41–43]. Previous studies have focused on the connection between SNPs of the VEGF gene and RSA; however, the results have been conflicting. There are several reasons for this controversy, such as diversities in sample size, country of origin, study designs, and statistical methods. A recent meta-analysis by Zhang et al. suggested that VEGF gene −634G/C and+936C/T polymorphisms were associated with the risk of RSA under specific genetic models[24]. However, those authors failed to assess the relationship between the other common polymorphisms in the VEGF gene and RSA risk. On the other hand, in recent years, several studies have reevaluated the connection between RSA and VEGF polymorphisms [24–27] and have raised the possibility of the association between RSA risk and more SNPs of the VEGF gene. The present meta-analysis aimed to update the previous meta-analysis as well as to provide a more comprehensive and reliable conclusion on the associations between 5 common functional polymorphisms in the VEGF gene and RSA susceptibility.

In our meta-analysis, 10 independent case-control studies were included with a total of 1,832 RSA patients and 2,271 healthy controls. When all the eligible studies were pooled into the meta-analysis, the results suggested that −1154G/A (rs1570360), +936C/T (rs3025039), −634G/C (rs2010963), and −583T/C (rs3025020) polymorphisms correlated with an elevated risk of RSA, indicating that these 4 polymorphisms may be risk factors for RSA. However, no statistically significant association was observed between −2578C/A (rs699947) and RSA risk. One possible reason for this pattern of results could be that rs1570360, rs3025039, rs2010963, and rs3025020 polymorphisms were more impactful than other SNPs on VEGF gene expression and protein production, thereby possibly explaining inter-individual differences in disease incidences of RSA. Furthermore, in the subgroup analysis by geographic position, significantly increased RSA risk was observed in non-Asian populations for rs1570360 polymorphism and Asian populations for rs3025039 polymorphism. A possible reason for geographic variation could be that great disparities in common SNPs in the VEGF gene that influence the risk of RSA are mostly due to genetic drift and natural selection[44]. The discrepancy could also be explained by the small sample size of some included studies, which may result in substantial errors from estimation. Population stratification and sample size are important issues to be concerned in human genetic surveys [45]. Thus, rs1570360 and rs3025039 polymorphisms may play various roles in RSA susceptibility in various geographic groups. The present meta-analysis of the relationship between the rs1570360 polymorphism and RSA risk differs from results previously reported by Zhang et al. [24]. This disagreement may be because the present study included 4 more studies[16,17,19,20] and excluded studies where diagnostic criteria of RSA patients were at least two consecutive spontaneous abortions[26,27]. However, our findings are partially consistent with the previous studies, indicating that VEGF genetic polymorphisms may be related to increased RSA risk and may be useful biomarkers for predicting individual susceptibility to RSA. And further functional studies to confirm the role of a putative SNP in RSA are still necessary.

Our meta-analysis has several limitations that should be taken into account. First, for some outcomes, sample size was relatively small, which may result in a lack of sufficient statistical power to estimate the association between VEGF genetic polymorphisms and RSA risk. Therefore, further investigations with larger sample size are still needed. Second, a meta-analysis is a retrospective study and may encounter recall or selection bias, thereby possibly influencing the reliability of our conclusions [46]. Third, when this analysis was carried out, we did not consider whether women had recurrent losses and never achieved a viable live birth successfully or if the loss(es) followed a successful pregnancy. There were also significant differences in control participants, especially in times of obstetric history. Without considering these disparities, analyses may be imprecise and valid correlations may go undetected. Finally, further evaluation of the potential value of these polymorphisms were limited due to lack of access to the original study data.

## Conclusion

Our meta-analysis reevaluates the relationship between VEGF genetic polymorphisms and RSA risk and demonstrates that −1154G/A (rs1570360), +936C/T (rs3025039), −634G/C (rs2010963), and −583T/C (rs3025020) polymorphisms in the VEGF gene are associated with susceptibility to RSA. These polymorphisms may be useful biomarkers for predicting individual susceptibility to RSA. Moreover, rs1570360 and rs3025039 polymorphisms may play various roles in RSA susceptibility in various geographic groups. Considering the limitations mentioned above, further well-designed studies with larger sample sizes should be performed to confirm our findings.

## Supporting Information

S1 Meta analysis on genetic association studies Checklist (DOCX)

S1 PRISMA Checklist (DOC)
